# Daily Cisplatin and Weekly Docetaxel versus Weekly Cisplatin Intra-Arterial Chemoradiotherapy for Late T2-3 Tongue Cancer: A Pilot and Feasibility Trial

**DOI:** 10.3390/medicina54040052

**Published:** 2018-07-30

**Authors:** Yuichiro Hayashi, Shuhei Minamiyama, Takashi Ohya, Masaki Iida, Toshinori Iwai, Toshiyuki Koizumi, Senri Oguri, Makoto Hirota, Mitomu Kioi, Masaharu Hata, Masataka Taguri, Kenji Mitsudo

**Affiliations:** 1 Department of Oral and Maxillofacial Surgery, Graduate School of Medicine, Yokohama City University 3-9 Fukuura, Kanazawa-ku, Yokohama, Kanagawa 236-0004, Japan; s.minamiyama@gmail.com (S.M.); marufuku33@gmail.com (T.O.); masaki.iida0316@gmail.com (M.I.); iwai104oams@yahoo.co.jp (T.I.); koizumi-tky@umin.ac.jp (T.K.); senri019@yokohama-cu.ac.jp (S.O.); mhirota@yokohama-cu.ac.jp (M.H.); kioi@yokohama-cu.ac.jp (M.K.); mitsudo@yokohama-cu.ac.jp (K.M.); 2 Department of Radiation Oncology, Graduate School of Medicine, Yokohama City University 3-9 Fukuura, Kanazawa-ku, Yokohama, Kanagawa 236-0004, Japan; hatahata@yokohama-cu.ac.jp; 3 Department of Biostatistics, Graduate School of Medicine, Yokohama City University 3-9 Fukuura, Kanazawa-ku, Yokohama, Kanagawa 236-0004, Japan; masataka.taguri@gmail.com

**Keywords:** oral cancer, radiotherapy, intra-arterial chemotherapy, head and neck cancer

## Abstract

*Background and objectives:* The aim of present study was to compare the treatment results of daily cisplatin (CDDP), weekly docetaxel (DOC) intra-arterial infusion chemotherapy combined with radiotherapy (DIACRT) regimen and weekly CDDP intra-arterial infusion chemotherapy combined with radiotherapy (WIACRT) for patients with tongue cancer. *Materials and Methods:* Between January 2007 and December 2016, a total of 11 patients treated with WIACRT and 45 patients treated with DIACRT were enrolled in the present study. In the DIACRT group, 25 patients had late T2, and 20 patients had T3. A total of nine patients had late T2 and two had T3 in WIACRT (*p* = NS). In DIACRT, the treatment schedule consisted of intra-arterial chemotherapy (DOC, total 60 mg/m^2^; CDDP, total 150 mg/m^2^) and daily concurrent radiotherapy (RT) (total, 60 Gy). In WIACRT, the treatment schedule consisted of intra-arterial chemotherapy (CDDP, total 360 mg/m^2^) and daily concurrent RT (total, 60 Gy). *Results:* The median follow-up periods for DIACRT and WIACRT were 61 and 66 months, respectively. The five-year local control (LC) and overall survival (OS) rate were 94.5% and 89.6% for the DIACRT group, and 60.6% and 63.6% for the WIACRT group, respectively. The LC rate and OS of the DIACRT group were significantly higher than those of the WIACRT group. As regards toxicities, no treatment-related deaths were observed during the follow-up periods in both groups. *Conclusions:* DIACRT was found to be feasible and effective for patients with tongue cancer and could become a new treatment modality.

## 1. Introduction

Intra-arterial chemoradiotherapy (IACRT) has become a promising treatment modality for advanced head and neck cancer in recent decades. Several studies were based on the method pioneered by Robbins et al. [1], which employs a four-weekly intra-arterial infusion of cisplatin (CDDP) to achieve a high-dose intensity directed into the tumor bed, with the simultaneous intravenous infusion of the CDDP neutralizing agent, sodium thiosulfate (STS). There are two methods of superselective catheterization for intra-arterial infusion in head and neck cancer. In one method, the catheter is inserted through the femoral artery using the Seldinger technique [2,3,4], and in the other, the catheter is inserted through the superficial temporal artery (STA) [5].

In our institution, intra-arterial infusion chemotherapy for oral cancer is performed via the STA and/or occipital artery (OA) for the purpose of avoiding neurological complications occasionally observed by the Seldinger method [2]. The main treatment protocol of chemoradiotherapy (CRT) consists of daily CDDP and weekly docetaxel (DOC) intra-arterial infusion (DIACRT) for the purpose of preventing treatment dropout due to four-weekly high-dose CDDP therapy adverse events [2,3,4,6].

Both weekly CDDP intra-arterial chemoradiotherapy (WIACRT) and our treatment regimen, DIACRT, are widely accepted for head and neck cancer in Japan. However, there are few studies comparing the treatment results of DIACRT and WIACRT for oral cancer patients. In the present study, we compared the treatment outcomes of DIACRT and WIACRT in patients with late T2-3 tongue cancer.

## 2. Materials and Methods

### 2.1. Study Design and Patients

**Figure 1:**
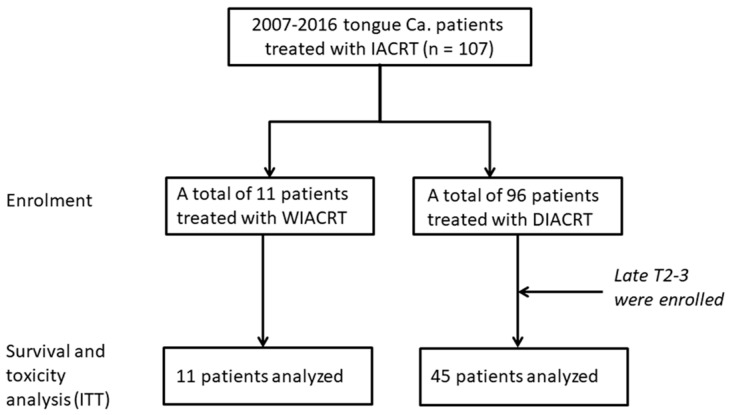
A Flowchart of the Study Design. All patients treated with weekly cisplatin (CDDP) intra-arterial chemoradiotherapy (WIACRT) were enrolled in the study (*n* = 11). A total of 45 patients with late T2-3 disease were selected from 96 patients treated with daily CDDP and weekly docetaxel (DOC) intra-arterial chemoradiotherapy (DIACRT) to get comparability between two groups.

This retrospective observational cohort study was performed in patients with tongue cancer (the anterior 2/3 of the tongue) treated with DIACRT or WIACRT at Yokohama City University Hospital (*n* = 107). In our institution, IACRT is performed for patients who refused surgery and WIACRT is performed for late T2 (measuring > 3 cm) and T3 patients, and DIACRT for late T2–T4 paients, especially for cases where the tumor invades the adjacent organ (buccal mucosa, floor of mouth, and lower gingiva) of the tongue. Between January 2007 and December 2016, a total of 11 patients were treated with WIACRT and 96 were treated with DIACRT. All 11 patients treated with WIACRT were enrolled in the present study. In the 96 patients treated with DIACRT, 45 patients had late T2-3 and 51 had T4a-4b. To assess the comparability of the two groups, a total of 45 patients with late T2-3 in the DIACRT group were enrolled in the present study. As a result, the 1:4 matched groups were created from the 96 patients treated with DIACRT to enhance the quality of statistical results [7]. A flowchart of the study design is shown in Figure 1. Specific inclusion criteria were as follows: ≥20 but <90 years of age; Eastern Cooperative Oncology Group performance status (ECOG-PS) of 0 or 1; pathological confirmation of squamous cell carcinoma of the tongue, with any tumor differentiation; adequate hematologic, hepatic, and renal functions (white blood cells, >3000/μL; neutrophils, >2000/μL; platelet count, >100,000/μL; hemoglobin, >9 g/dL; AST and ALT, <3 times the normal upper limit; total bilirubin, <1.5 mg/dL; and creatinine clearance, >60 mL/min); and life expectancy of ≥3 months.

This study was approved by the local institutional review board of Yokohama City University Hospital (No. B110707032). Written informed consent was obtained from all patients and the investigators followed recommendations of the Helsinki Declaration.

### 2.2. Clinical Response Evaluation

Clinical response was evaluated in all patients using imaging modalities, namely PET/CT and enhanced MRI, three months after the completion of the treatment schedule. Clinical response was judged according to the Response Evaluation Criteria in Solid Tumors. If there were residual primary tumors and metastatic lymph nodes after treatment, salvage surgery including neck dissection was performed.

### 2.3. Radiotherapy

Radiotherapy (RT) was performed concurrent with IACRT five times a week with a fraction size of 2 Gy using a 6 MV linear accelerator for both WIACRT and DIACRT groups. The gross tumor volume (GTV) was defined as any visible evidence of disease on physical examination or on any imaging modality, including contrast-enhanced CT and MRI examination. The clinical target volume (CTV) was defined as GTV with a 5-mm margin in all directions to cover microscopic disease. The CTV was expanded by 5 mm in all directions to create the planning target volume (PTV) for setup uncertainty. For patients with no cervical lymph node metastases, the irradiation field was set up to cover the primary lesion and prophylactically the level I-III lymph node regions as the CTV. For patients with cervical lymph node metastases, the irradiation field was set up to cover the primary tumors and the ipsilateral (levels I–IV for N1) cervical lymph node areas, including lymph node metastases, as the CTV. After a total dose of 40 Gy had been delivered to the initial field, an additional 20 Gy was delivered to the primary tumors and metastatic lymph nodes within the shrunken field. A boost of up to 10 Gy (total dose, 70 Gy) was allowed in cases of persistent clinical residual tumor or to compensate for potential treatment delay (over five days) during the treatment course.

### 2.4. Chemotherapy

**Figure 2:**
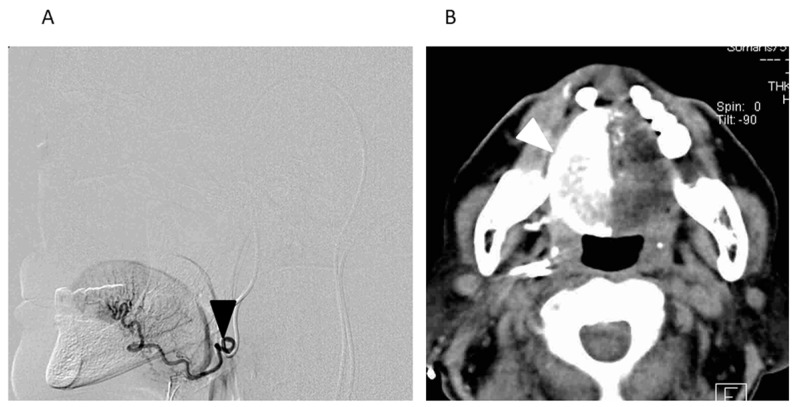
A Digital Subtraction Angiogram (DSA) and an Axial View of the Angio-CT Image through Retrograde Intra-arterial Infusion (Right Tongue Cancer: T3N0M0). (**A**) A DSA of the right lingual artery (LA). The catheter was selectively inserted into the right LA via the occipital artery (black arrowhead: the tip of the catheter). (**B**) An axial view of the angio-CT image after the infusion of a small amount of contrast medium through the catheter. Tumor staining of the right side of the tongue is seen through the right LA (white arrowhead).

Catheterization via the STA and/or occipital artery was performed prior to IACRT following past reports [5,8]. The tongue is usually fed by the lingual artery (LA); therefore, the tip of the catheter was selectively inserted into the LA. In cases where the tumor had spread to the lingual side of the lower gingiva or the floor of the mouth, catheters were inserted into the facial artery (FA) in addition to the LA. Moreover, when the lesion involved the contralateral side beyond the median line, another catheter was inserted in the contralateral side. After catheterization, the perfusion area of the anticancer agent was confirmed by digital subtraction angiography and angio-CT (Figure 2). Angio-CT was performed with slow infusion via a catheter to determine whether the anticancer agents delivered via intra-arterial infusion permeated the entire tumor.

**Figure 3:**
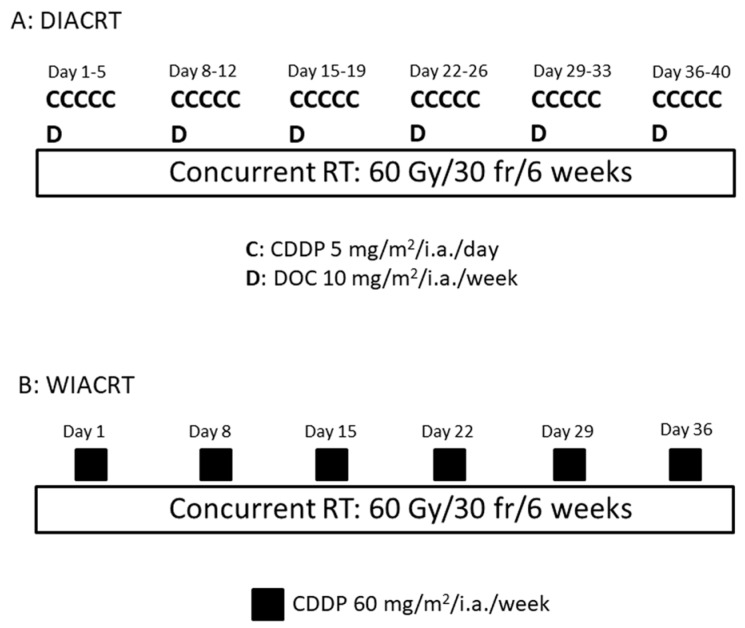
Treatment Schedule of DIACRT and WIACRT. In daily cisplatin (CDDP) and weekly docetaxel (DOC) intra-arterial chemoradiotherapy (DIACRT), the treatment schedule consisted of intra-arterial chemotherapy (DOC, total 60 mg/m^2^; CDDP, total 150 mg/m^2^) and daily concurrent radiotherapy (RT) (total, 60 Gy) for six weeks. In weekly CDDP intra-arterial chemoradiotherapy (WIACRT), the treatment schedule consisted of intra-arterial chemotherapy (CDDP, total 360 mg/m^2^) and daily concurrent RT (total, 60 Gy) for six weeks.

The anticancer agent was injected in a bolus through the intra-arterial catheter concurrent with RT. In the DIACRT group, the total dose of CDDP was 150 mg/m^2^ (5 mg/m^2^/day, five times a week for six weeks) and that of DOC was 60 mg/m^2^ (10 mg/m^2^/week for six weeks). Sodium thiosulfate (STS) (1 g/m^2^) was administered intravenously immediately after intra-arterial infusion of CDDP. The DIACRT regimen was determined according to other studies [9,10,11]. In the WIACRT group, CDDP was administered as an intra-arterial infusion at doses of 30–60 mg/m^2^ once a week for six weeks. STS was also administered intravenously at 2 g/m^2^ immediately after intra-arterial infusion of CDDP (Figure 3). The weekly CDDP dose of WIACRT was determined according to the study of Fuwa et al. [12].

### 2.5. Evaluation of Toxicity

Acute and late toxicities were evaluated according to the National Cancer Institute’s Common Terminology Criteria for Adverse Events version 4.0 (CTCAE v4.0). The evaluation categories for acute toxicities were blood cell counts, acute kidney injury (AKI), nausea/vomiting, catheter-related infection (CRI), febrile neutropenia (FN), oral mucositis, radiation dermatitis, dysphagia, and neurologic toxicity. For late toxicities, xerostomia and osteoradionecrosis (ORN) were evaluated. The two treatment groups were compared using the chi-square test for each adverse event.

### 2.6. Statistical Analysis

All statistical analyses were performed using IBM SPSS Statistics version 20 (SPSS Inc., Chicago, IL, USA). The two treatment groups (WIACRT and DIACRT) were compared using the chi-square test for categorical variables (age, sex, ECOG-PS, TNM status, and treatment delivery). The overall survival (OS) and local control (LC) rates were calculated using the Kaplan-Meier method, and the log-rank test was used to compare the survival curves. Events were measured from the start of treatment. All statistical tests were two-sided and based on an intent-to-treat manner, and the significant level was set at 0.05.

## 3. Results

### 3.1. Patients’ Characteristics

Patients’ characteristics are shown in Table 1. The median age was 60 years (range, 36–72 years) in the DIACRT group and 59 (range, 40–78) in the WIACRT group. In the DIACRT group, 25 patients (55.6%) had late T2, and 20 patients (44.4%) had T3. A total of nine patients (81.8%) had late T2 and 2 (18.2%) had T3 in WIACRT. In patients’ characteristics, there was no significant difference in categorical variables between the two groups.

### 3.2. Treatment Delivery

In the WIACRT group, three patients discontinued treatment due to severe AKI (*n* = 1) and laryngeal edema (*n* = 2). The completion rate of the WIACRT regimen was 73%. In these patients, salvage surgery was performed immediately. In the WIACRT group, the median total dose of RT was 60 Gy (range, 4–60 Gy). During the treatment period, two patients experienced treatment delay due to FN and AKI. As regards intra-arterial infusion chemotherapy, the median cumulative dose of CDDP was 360 mg/m^2^ (range, 60–360 mg/m^2^).

In the DIACRT group, a total of 43 patients (95.6%) completed the whole treatment course. A total of two patients (4.4%) discontinued treatment due to catheter-related infection. The median total dose of RT for all patients treated with DIACRT was 60 Gy (range, 50–70 Gy). Three patients (6.7%) had treatment delay due to neutropenia. The median cumulative CDDP and DOC doses were 150 mg/m^2^ (range, 135–175 mg/m^2^) and 60 mg/m^2^ (range, 60–70 mg/m^2^), respectively. A cumulative dose of CDDP in WIACRT was significantly higher than that of DIACRT (*p* = 0.0023) (Table 1).

### 3.3. Response and Survival

**Figure 4:**
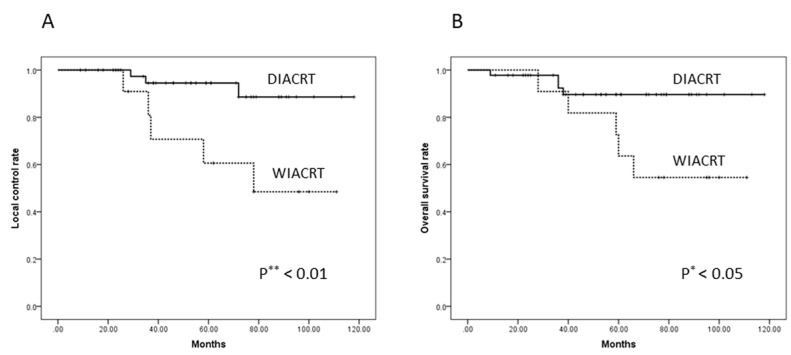
(**A**) The five-year Local Control (LC) and (**B**) Overall Survival (OS) Rates of the DIACRT and WIACRT Groups. The five-year LC and OS were 94.5% and 89.6% for the DIACRT group, and 60.6% and 63.6% for the WIACRT group, respectively. The LC and OS rate in the DIACRT group were significantly higher than those in the WIACRT group (*p* = 0.007 and 0.027, respectively). Abbreviations: OS, overall survival; LC, local control.

The median follow-up periods for DIACRT and WIACRT were 61 and 66 months, respectively. A complete response was achieved in eight of 11 patients (72.7%) in the WIACRT group and 43 of 45 (95.6%) in the DIACRT group. During the follow-up period, local recurrence occurred in four patients (36.4%) in the WIACRT group and three (6.7%) in the DIACRT group. Among the four patients with local recurrence in the WIACRT group, two (18.2%) underwent salvage surgery. Similarly, three patients (6.7%) in the DIACRT group underwent salvage surgery. In the WIACRT group, one patient (9.1%) died of bleeding from a recurrent primary tumor, and four (36.4%) died of lung metastases during the follow-up period. In the DIACRT group, three patients (6.7%) died of lung metastases and one patient (2.2%) died of local relapse during the follow-up period. The five-year LC and OS were 94.5% and 89.6% for the DIACRT group, and 60.6% and 63.6% for the WIACRT group, respectively. The LC and OS rate in the DIACRT group were significantly higher than those in the WIACRT group (*p* = 0.007 and 0.027, respectively; Figure 4A,B).

### 3.4. Toxicities

Acute and late toxicities are summarized in Table 2. In the DIACRT group, toxicities of grade 3 or higher included neutropenia in three patients (6.7%), anemia in three (6.7%), mucositis in 32 (71.1%), dermatitis in 11 (24.4%), and dysphagia in 30 (66.7%). There was no significant difference in categorical variables between the two groups. However, grade 4 AKI and grade 3 ORN were only seen in the WIACRT group. A patient who experienced grade 3 ORN in the WIACRT group needed surgical treatment. In addition, there were no transient or persistent central nervous system complications and treatment-related deaths during the treatment and follow-up period.

## 4. Discussion

In recent decades, CRT using CDDP is the optimal treatment modality for patients with an inoperable region or those who refuse surgical treatment. A high-dose bolus of intravenous CDDP (100 mg/m^2^) every three weeks concurrent with RT is the most widely used regimen; however, this high dose of CDDP is associated with significant acute and late toxicities, and the completion rate of the treatment also remains a challenge [13,14,15]. Therefore, various doses of CDDP schedules have been studied, such as 100 mg/m^2^ three-weekly, 35–60 mg/m^2^ weekly, and 6 mg/m^2^ daily [13,14,15,16,17,18,19,20]. In the present study, we employed a relatively low dose CDDP regimen for both WIACRT (30–60 mg/m^2^ at weekly) and DIACRT (5 mg/m^2^ at daily). Additionally, treatment-related hematological toxicities were relatively mild in both the WIACRT and DIACRT groups. Rades et al. [20] also compared the treatment results of different lower-dose programs of CDDP used in concurrent CRT protocols for locally advanced head and neck cancer patients and reported that intravenous daily administration of CDDP may be preferable than weekly administration for head and neck cancer patients. The DIACRT regimen employed the daily administration of CDDP, and therefore achieved better treatment outcomes than the WIACRT.

In the present study, DOC was also administered weekly in addition to daily CDDP infusion in the DIACRT group. Compared with the high recurrence rate in the WIACRT group, the DIACRT group demonstrated a significantly high LC rate. Both DOC and CDDP were used owing to their different mechanisms of action as either a cytotoxic agent or a radiosensitizer. Treatment with DOC followed by CDDP demonstrated a synergistic effect on cell survival inhibition, with greater intracellular platinum accumulation compared to that observed after treatment with CDDP followed by DOC, and DOC improved the multidrug resistance induced by a single treatment with CDDP [21]. Furthermore, according to the report by Yabuuchi et al., the combined use of two different anticancer agents achieved better clinical outcomes for head and neck cancer than single use [22]. In the present study, it was possible that weekly DOC infusion in addition to daily CDDP contributed to good treatment results. Obtaining statistically significant LC, the DIACRT was also concluded to be a more preferable regimen than WIACRT in terms of organ preservation for the primary lesion.

In the present study, we employed IACRT, not systemic CRT, for tongue cancer patients as a definitive therapy and demonstrated good LC and OS, especially in the DIACRT group. It is a well-known fact that the radiation sensitivity of an oral cavity is lower than other head-and-neck regions [23,24]. Moreover, advanced oral cancers might have a worse response to systemic CRT than other head and neck cancers. Iqbal et al. [24] reported on the treatment outcomes of head and neck cancer treated with CRT using weekly intravenous CDDP (40 mg/m^2^) for five weeks (*n* = 122). They investigated the prognostic factors that affected the survival and revealed that oral cancer had the worst prognosis. Moreover, Fuwa et al. [25] reported that systemic chemotherapy was not a significant factor for survival in the combination therapy of systemic and intra-arterial chemotherapy concurrent with RT for locally advanced oral cancer. With these findings, it can be presumed that systemic chemotherapy is not effective for advanced oral cancer.

Selective injection of anticancer agents into the tumor-feeding artery is an effective method to achieve higher doses of anticancer agents in the tumors with less systemic toxicities than intravenous chemotherapy [1,2,3,4,5,6,25]. Fuwa et al. [25] reported that weekly doses of superselective continuous intra-arterial carboplatin via the STA and concurrent RT could be delivered safely with good three-year LC and OS rates for locally advanced oral carcinoma.

Several studies of low-dose weekly intra-arterial chemotherapy using CDDP for head and neck cancer have been reported and demonstrated good treatment results [26,27,28]. Takayama et al. [28] reported on the treatment results of advanced tongue cancer treated with a weekly intra-arterial CDDP infusion via the STA concurrent with RT and proton therapy (The 3-year OS: 87%). However, most of the local failures in the WIACRT group were seen three to five years after treatment was completed in the present study. A long-term follow-up is necessary after CRT because oral cancer has a high recurrence rate compared with other head neck cancer [29]. In the present study, the DIACRT regimen was more useful in that its LC and OS rate were significantly higher than those of WIACRT for patients with tongue cancer, with a median follow-up of ≥5 years.

In the present study, no neurological complications were observed in both the DIACRT and WIACRT groups. This was presumably because catheterization through the STA does not pass through the common carotid artery [5]. Furthermore, the STA approach is technically simple and probably an easier method of inserting a catheter into the target artery selectively than the transfemoral approach [30].

It was also important to note that the completion rate of the DIACRT regimen was higher than that of WIACRT in the present study. A high dropout rate (27%) was seen in the WIACRT group during the treatment period. In this respect, the DIACRT regimen would be safer and more feasible than WIACRT. DIACRT was generally tolerated with few severe adverse events including renal failure and fever. This was because the median cumulative CDDP dose in DIACRT was relatively lower compared with that of general systemic chemotherapy and WIACRT.

Almost all patients experienced mucositis over grade 2 as an acute toxicity in both DIACRT and WIACRT groups. Severe mucositis and dysphagia were also inevitable in previous IACRT studies [2,6,28,30]. In this study, percutaneous endoscopic gastrostomy (PEG) was performed on most patients in the DIACRT and WIACRT groups at an early stage of treatment. It was supposed that the proper use of PEG tubes for feeding prevented the deterioration of nutritional status. We recognize the limitations of the present study. This is a retrospective pilot and feasibility study, and therefore a prospective multicenter clinical trial to evaluate the efficacy of DIACRT for oral cancer patients should be conducted in the future.

## 5. Conclusions

DIACRT was found to be more feasible and effective for patients with tongue cancer compared with WIACRT.

## Figures and Tables

**Table 1:** Patients’ characteristics and treatment delivery.

		Treatment Type		
*Characteristics*	Total No.	DIACRT	WIACRT	*p* Value
No. of patients	56	45	11	
Median age, years (range)	59 (36–78)	60 (36–72)	59 (40–78)	0.332
Gender				
Male	29	22	7	0.612
Female	27	23	4	
Performance status (ECOG)				
0	53	43	10	0.787
1	3	2	1	
T classification				
Late T2	34	25	9	0.392
T3	22	20	2	
N classification				
N0	42	34	8	0.215
N1	14	11	3	
Stage				
II	27	20	7	0.553
III	29	25	4	
Tumor differentiation				
Low grade	5	4	1	0.275
Moderate grade	23	19	4	
High grade	28	22	6	
*Treatment delivery*				
Median RT dose (Gy) (range)		60 (50–70)	60 (4–60)	0.422
Cumulative dose of CDDP (mg/m^2^) (range)		150 (135–175)	360 (60–360)	0.0023

Note: Values represent number of patients, except as otherwise stated. Abbreviations: ECOG, Eastern Cooperative Oncology Group; RT, radiotherapy; Gy, gray; CDDP, cisplatin.

**Table 2:** Acute and late toxicities over Grade 2 (CTCAE v4.0).

	DIACRT (*n* = 45)				WIACRT (*n* = 11)				
Toxicities	G 2	G 3	G 4	G 5	G 2	G 3	G 4	G 5	*p* Value
***Acute***									
Neutropenia	8	3	0	0	4	1	0	0	0.213
Thrombocytopenia	8	0	0	0	2	0	0	0	0.665
Anemia	8	3	0	0	4	1	0	0	0.112
AKI	3	0	0	0	4	0	1	0	0.364
Nausea/vomiting	2	0	0	0	4	0	0	0	0.153
CRI	0	4	0	0	0	0	1	0	0.711
FN	2	0	0	0	0	1	0	0	0.104
Mucositis	13	32	0	0	5	5	0	0	0.222
Dermatitis	28	11	0	0	5	4	0	0	0.412
Dysphagia	15	30	0	0	5	5	0	0	0.109
***Late***									
xerostomia	13	0	0	0	7	0	0	0	0.193
ORN	9	0	0	0	4	1	0	0	0.207

Abbreviations CTCAE v 4.0: Common Terminology Criteria for Adverse Events version 4.0 G, Grade; AKI, acute kidney injury; CRI, catheter related infection; FN, febrile neutropenia; ORN, osteoradionecrosis.
